# PEEK and CFR-PEEK as alternative bearing materials to UHMWPE in a fixed bearing total knee replacement: An experimental wear study

**DOI:** 10.1016/j.wear.2016.12.010

**Published:** 2017-03-15

**Authors:** Claire L. Brockett, Silvia Carbone, John Fisher, Louise M. Jennings

**Affiliations:** Institute of Medical and Biological Engineering, University of Leeds, UK

**Keywords:** PEEK, CFR-PEEK, Knee replacement, Wear

## Abstract

New bearing materials for total joint replacement have been explored as the need to improve longevity and enhance performance is driven by the changing demands of the patient demographic. Carbon-reinforced PEEK has demonstrated good wear characteristics in experimental wear simulation in both simple geometry pin-on-plate studies and in total hip joint replacement. Carbon reinforced PEEK CFR-PEEK has the potential to reduce tibial insert thickness and preserve bone in the knee.

This study investigated the wear performance of PEEK and CFR-PEEK in a low conformity total knee replacement configuration. Custom-made flat inserts were tested against cobalt-chromium femoral bearings in a knee wear simulation for a period of three million cycles. Wear was assessed gravimetrically at intervals throughout the study.

The wear rates of both PEEK and CFR-PEEK were very high and almost two orders of magnitude higher than the wear rate of UHMWPE under comparable conditions. Evidence of mechanical failure of the materials, including surface cracking and delamination was observed in both materials.

This study highlights that these materials may not be suitable alternatives for UHMWPE in low-conformity designs.

## Highlights

• Study investigated wear of PEEK and CFR-PEEK as alternative to PE in TKR.
• Very high wear rates observed for both PEEK and CFR-PEEK.
• Wear rates two orders of magnitude higher than UHWMPE under same conditions.
• Evidence of mechanical failure of PEEK and CFR-PEEK materials.

## 1 Introduction

Total joint replacement has long been considered the intervention of choice for patients with late-stage arthritis of the knee. Millions of patients globally have benefited from reduction in pain and increased joint mobility through total knee replacement (TKR), and clinical survivorship is now above 90% at ten-year follow-up [1], [2]. Despite good clinical outcomes, changing patient demographics and expectations continue to drive the need for improved performance and longevity [3], [4].

Wear of the polyethylene bearing, in both fixed and mobile bearing knee replacements, continues to be a significant factor in the long-term performance of TKR [5]. Historically, failure was often associated with high contact stress, poor oxidative stability and inadequate mechanical properties of the polyethylene bearing leading to fatigue failure and material delamination [6], [7]. The perceived need to reduce contact stress within total knee replacement design led to the introduction of highly conforming, low contact stress designs. Whilst these implant geometries indeed reduced contact stress at the articulating surface, the increased conformity tended to lead to over-constraint in the joint, and risk of secondary stresses occurring within the inserts resulting in mechanical failure [8]. Furthermore, more recent experimental studies and wear theory highlight that lower conformity bearings yield lower wear rates due to the reduced contact area and sliding distance in fixed bearing knee replacements [9], [10].

Developments over the last decade have introduced changes in manufacturing and sterilisation methods, as well as advancements in material technology enhancing the mechanical properties, oxidative stability or wear performance of polyethylene. However, there appears to be an apparent trade-off between each of these factors, with increasing cross-linkage (proposed to enhance wear performance) associated with a decrease in fatigue resistance [11], [12]. There has been some exploration of alternative polymers to replace polyethylene in knee replacement, including the use of poly-ether-ether-ketone (PEEK) and carbon-fibre reinforced PEEK (CFR-PEEK). In most cases, the carbon fibres within the CFR-PEEK materials are small, chopped fibres and generally have no specific orientation within the PEEK matrix.

Both PEEK and CFR-PEEK materials have been investigated, through laboratory based studies, as alternative bearing materials against metallic and ceramic counterfaces, as well as in self-mating couples. Simple configuration pin-on-plate studies, with PEEK or CFR-PEEK pins articulating against cobalt-chromium, zirconia-toughened alumina ceramic and polymeric (typically UHMWPE or a self-mating combination) plates have generally demonstrated CFR-PEEK to have a wear performance comparable to or better than conventional polyethylene materials [13], [14], [15]. Whilst PEEK does not appear to demonstrate equivalent wear performance against hard bearings, pin on plate studies have indicated it may be a suitable material for articulation with another polymer [14], [16]. More recently, a study of PEEK and CFR-PEEK against hard counterfaces has indicated that these materials exhibit a contact-pressure wear response, highlighting the need for further testing in full joint configuration to determine performance under relevant conditions [17].

Total joint simulation has investigated the use of CFR-PEEK in place of polyethylene in conforming partial knee replacements, total hip replacements and a horse-shoe shaped tissue sparing cup design. The hip wear simulation studies, with both conventional and horse-shoe cups, demonstrated favourable wear performance of CFR-PEEK when studied with ceramic femoral heads, compared with conventional polyethylene [18], [19], [20]. The conforming partial knee replacement experimental studies indicated CFR-PEEK to have an equivalent or lower wear performance than the standard bearing arrangement [21], [22]. The potential wear advantage of CFR-PEEK bearings in total joint replacement appear to have been more clearly demonstrated in ceramic on CFR-PEEK hip replacements to date [18].

CFR-PEEK has clearly demonstrated some potential as an alternative bearing material to UHMWPE in total joint replacement, but further analysis is indicated from the preliminary pin-on-plate studies to assess the performance in a total knee replacement configuration. Polyethylene has demonstrated very low wear performance in a low conformity implant, where the contact stress was relatively high [9]. The aim of this study was to investigate the performance of PEEK and CFR-PEEK in a low conformity fixed bearing total knee replacement. Simple configuration studies have indicated a potential elevation in wear with increased contact pressure for PEEK and CFR-PEEK materials, therefore the hypothesis of this study was that both materials would exhibit higher wear than UHMWPE under comparable conditions.

## 2 Materials

The wear of PEEK and CFR-PEEK materials as a substitute for polyethylene was investigated in a fixed bearing knee configuration. PEEK (Optima Natural, Invibio) and CFR-PEEK (PEEK Optima Wear performance (identified as Motis when produced for this study), Invibio) were injection moulded to form flat rectangular tibial plates, of 15mm thickness. The plates were manufactured to give an equivalent thickness to the (insert plus tibial tray) of a commercially available fixed bearing total knee replacement. The thickness was derived by measuring the tibial tray with 10 mm thick curved insert of a clipped in position at the dwell point of the condyles. The orientation of the pitch-based carbon fibres (30% fill by weight) within the CFR-PEEK plates was not controlled during the manufacturing process, it was therefore expected the fibres would be randomly oriented throughout the PEEK matrix. The plates had 5mm counter-sunk holes drilled in the corners to allow them to be clamped to the tibial fixtures during the study to reduce the potential for back-side wear. All samples were placed in deionised water for a minimum of 12 weeks, in order for the fluid uptake to stabilise [18]. Previous studies have demonstrated CFR-PEEK materials to take a longer period than polyethylene to reach an equilibrated fluid absorption level to be achieved prior to the commencement of the wear study.

Both materials were tested against femoral bearings to replicate a fixed bearing total knee replacement configuration. The femoral components were commercially available bearings in current clinical use, a Sigma Cruciate Retaining design (DePuy International, UK), manufactured from a cobalt-chromium-molybdenum alloy. The same femoral bearings were used for both studies. Surface analysis was undertaken between studies to ensure the surface roughness of femoral components had not significantly altered, and therefore would not impact on the subsequent wear study.

## 3 Methods

**Fig. 1:**
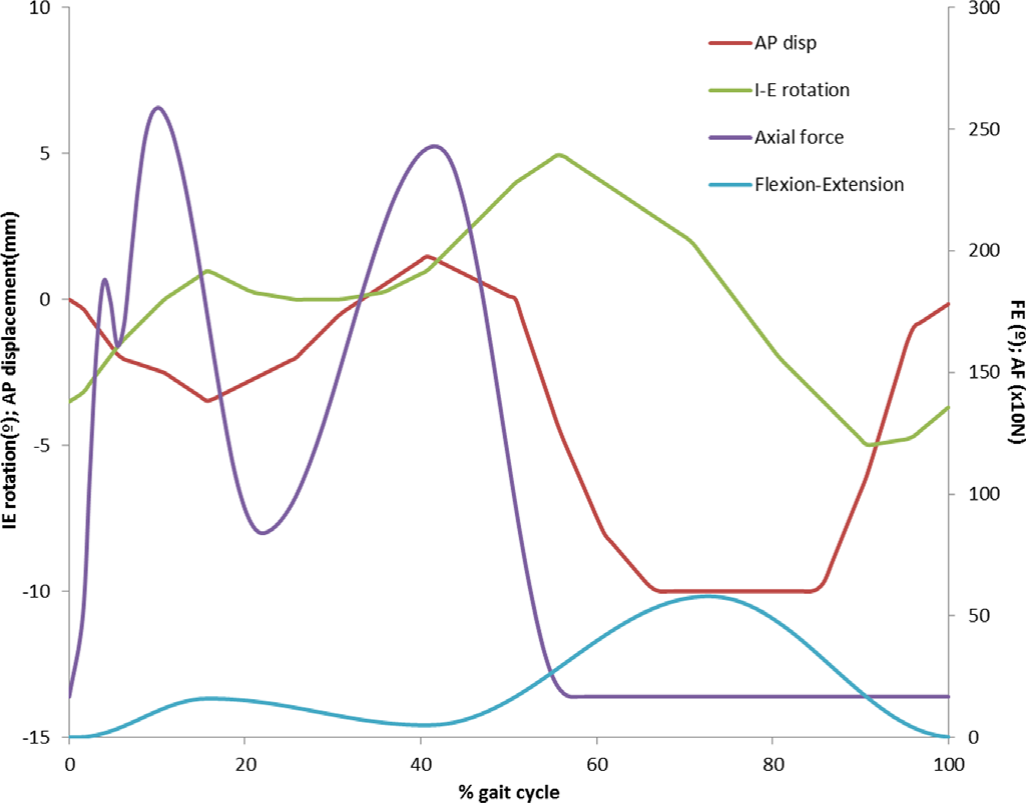
Input profile for wear simulator. (Note AP displacement and rotation are driven on the tibial tray, therefore –ve displacement is indicative of the contact between the femoral component and tibial tray moving posteriorly).

Two studies were conducted using the Leeds ProSim six station force/displacement controlled knee simulator (Simulator Solutions, UK). Four axes of motion were controlled (Fig. 1), with a peak load of 2.6 kN applied axially, and flexion-extension of 0–58° as defined in ISO14243-3 [23]. A ‘high-kinematic’ condition was used for both studies, with an anterior-posterior translation of 0–10 mm applied to the tibial bearing [24]. The tibial rotation, based on the natural kinematics of the knee, was set at ±5° [25]. The simulator was run under ‘displacement-control’, meaning both the rotation and displacement were controlled by displacement parameters. Abduction/adduction was allowed but not controlled. Six sets of femoral bearing and polymer tibial plates were tested for each material, mounted anatomically in each station. In order to eliminate station specific differences the samples were moved around the stations every million cycles [26].

The bearings were tested for three million cycles (Mc) under high kinematic conditions. The simulator was run at a frequency of 1 Hz. The lubricant used was newborn calf serum, diluted to 25%, supplemented with 0.03% (v/v) sodium azide to retard bacterial growth, and was changed every 0.33 Mc. Wear was determined gravimetrically through measurements of the inserts following the twelve-week soak period, and at measurement intervals throughout the study. A Mettler AT201 (Mettler-Toledo, USA) digital microbalance, which had a resolution of 0.01 mg, was used for weighing the bearing inserts. The volumetric wear was calculated from the weight loss measurements, using a density for CFR-PEEK of 1.42 g/cm^3^ and for PEEK of 1.3 g/cm^3^, using unloaded soak controls to compensate for moisture uptake [27]. The soak control samples were immersed in 25% bovine serum, as prepared for the test samples, and this serum was also changed every 0.33 Mc.

Digital images of the wear scars on the inserts at the completion of the study were obtained by capturing the image on a Kodak DX6490 digital camera. Surface roughness measurements of the femoral bearings were recorded at the start and completion of each study to examine the changes in roughness over the duration of the study. A contacting profilometer (Talysurf, Taylor Hobson, UK) was used and a least-squares arc form removal, and a Gaussian filter of 100:1 bandwidth, with a cut-off of 0.8 mm was applied to the femoral bearings. The same contacting profilometer was used to measure the penetration depth of the wear scars in the PEEK and CFR-PEEK inserts. Linear measurements were taken across the wear scars in a medial-lateral direction using a 2 µm-tipped stylus. Measurements were performed at a rate of 1 mm/s and measurements were recorded at 0.25 mm intervals in the anterior-posterior direction. The area measured was determined by the final wear scar area, with 3 mm of unworn bearing surface captured within each sample measurement to permit levelling and form-fitting. The maximum penetration depth for each insert was identified from the 3D data.

Scanning electron microscopy (back scattered electron (BSE)) was completed on the PEEK and CFR-PEEK inserts at completion of the study (EVO MA15-Smart SEM). SEM images were taken at 20 keV, with an estimated resolution of 0.5 µm.

Statistical analysis on the gravimetric wear data and wear scar depth was performed using a dependent samples t-test, and significance taken at p<0.05.

## 4 Results

**Fig. 2:**
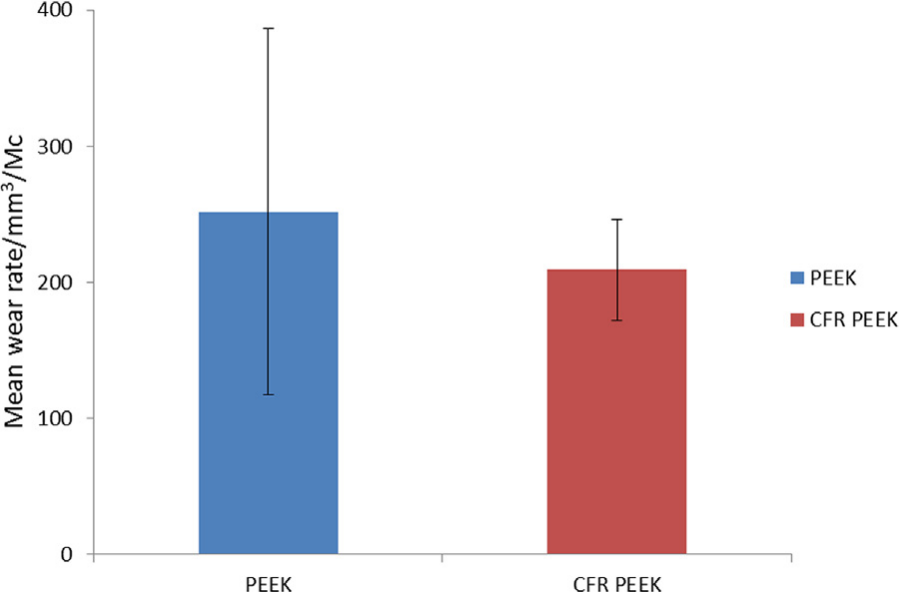
Mean wear rates (±95% confidence limits).

The mean wear rates for the PEEK and CFR-PEEK bearings after three million cycles of wear testing are shown in Fig. 2 (with 95% confidence limits indicated). The mean wear rates of the PEEK inserts was 252±159 mm^3^/Mc, and the mean wear rate for the CFR-PEEK inserts was 209±37 mm^3^/Mc. There was no statistically significantly difference between the wear rates of the materials (p>0.05). The PEEK material appeared to have much greater inter-specimen variability in wear rate compared with the CFR-PEEK material, as evidenced by the large confidence limits. Both materials exhibited linear wear behaviour throughout the study.

**Fig. 3:**
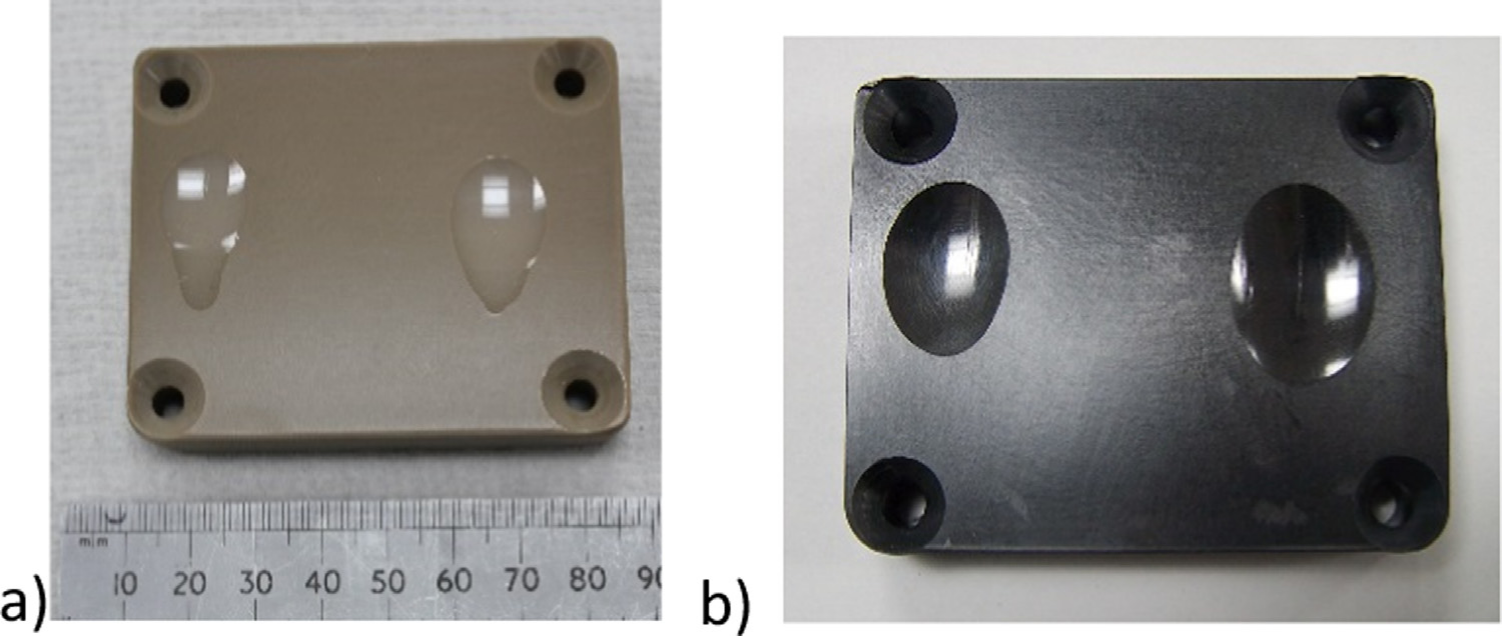
Wear scar areas for (a) PEEK and (b) CFR PEEK at completion of each study.

**Fig. 4:**
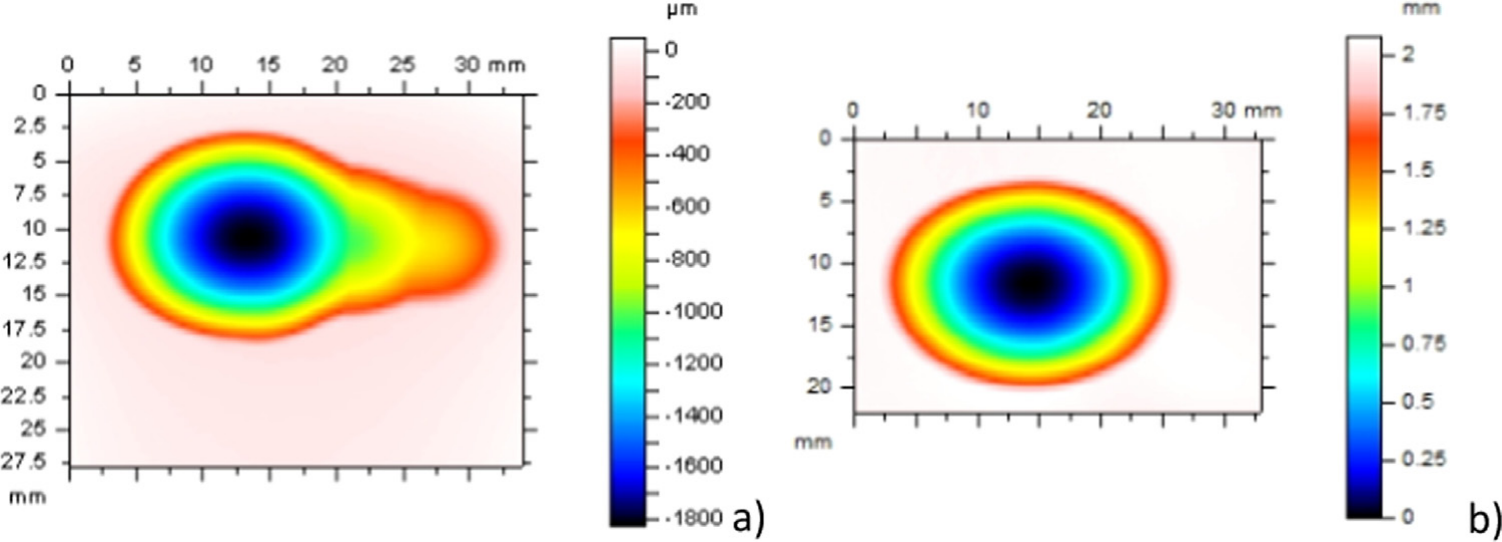
Example 3D wear scar traces for a) medial scar PEEK insert b) medial scar CFR-PEEK insert.

At the completion of each test large, deep wear scars were clearly visible on all insert surfaces (Fig. 3). 3D Talysurf measurements were obtained to determine the maximum penetration depths for each bearing. The largest penetration was shown in the ‘medial’ wear scar for both materials (Fig. 4). The mean peak scar depths in the medial scars were 1.86±0.59 mm and 2.08±0.13 mm in the PEEK and CFR-PEEK materials respectively. The mean peak scar depths on the lateral side were 1.53±0.38 mm and 1.54±0.14 mm for the PEEK and CFR-PEEK materials. There was no significant difference between the peak scar depth of the materials in either medial or lateral scars (p>0.05).

The femoral bearings were reused between the PEEK and CFR-PEEK studies. At completion of the PEEK study, the femoral bearings showed very light linear scratches, and the mean surface roughness did not change significantly over the course of the study (Ra of 0.05±0.02 µm pre-test and 0.07±0.03 µm post-test; (p>0.05)). Following completion of the CFR-PEEK wear study, the femoral bearings appeared to be highly scratched and the mean surface roughness had increased to 0.13±0.03 µm.

**Fig. 5:**
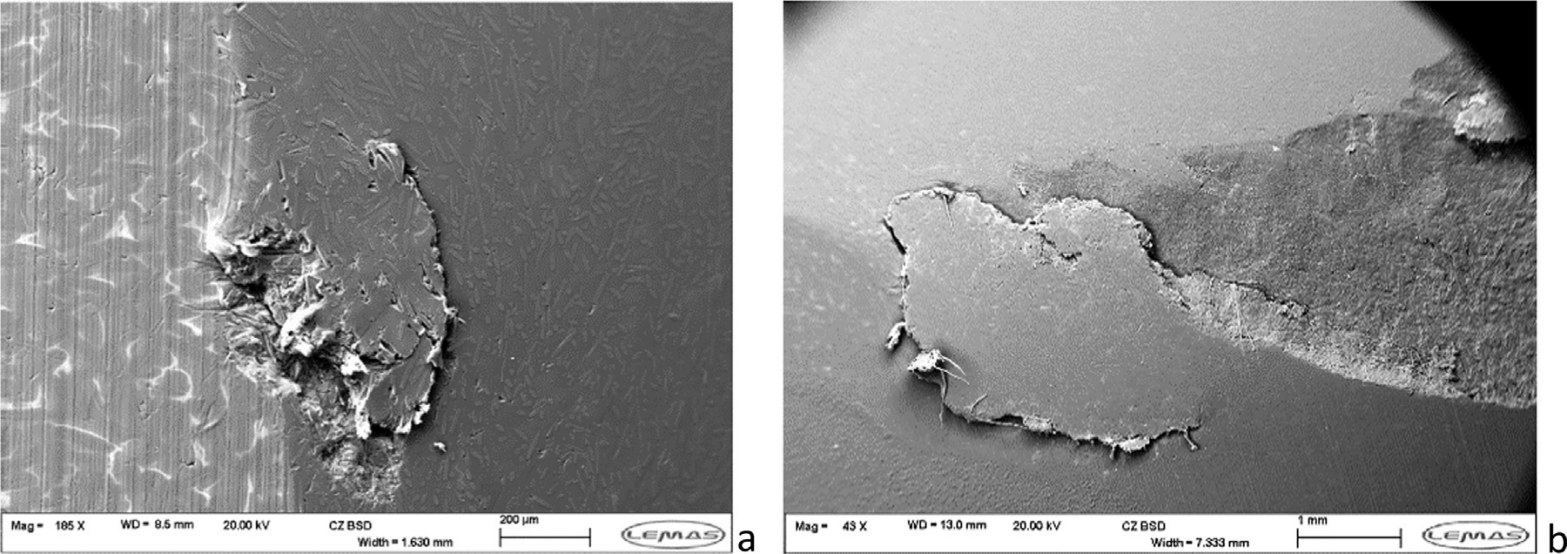
Examples of surface failure identified through SEM imaging of CFR-PEEK inserts (a) delamination and fibre pull out (b) cracking and delamination.

**Fig. 6:**
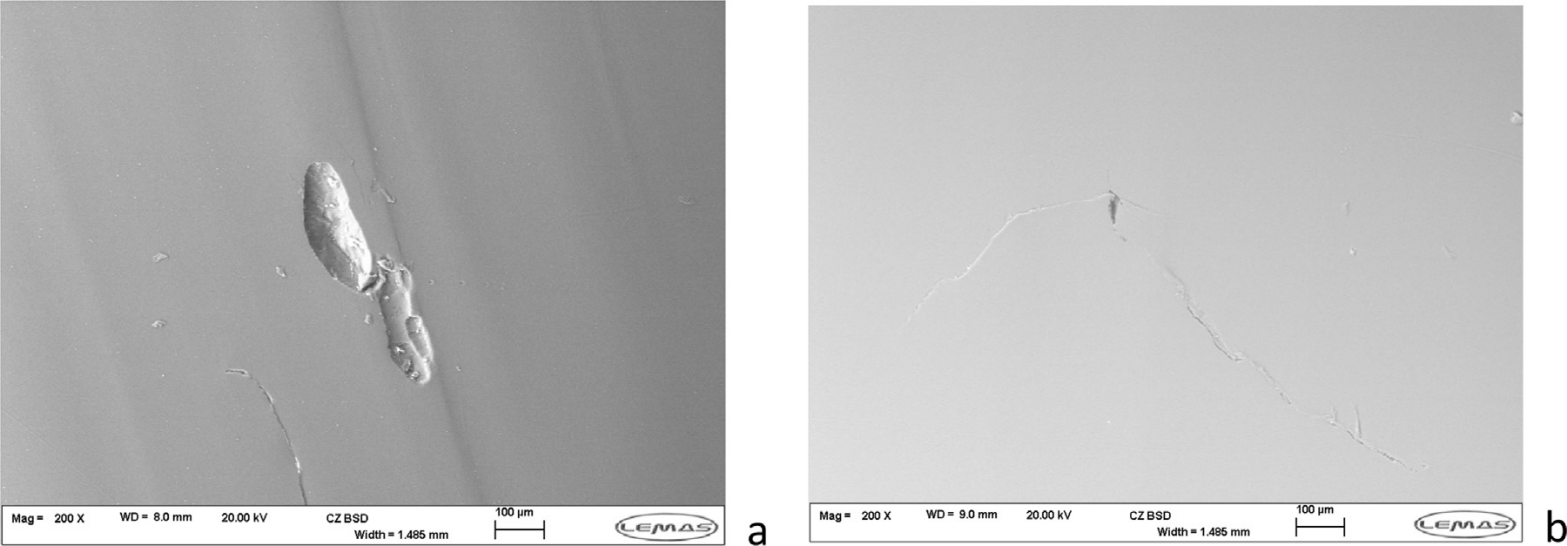
Examples of surface failure identified through SEM imaging of PEEK inserts (a) pits (b) cracking.

Visual inspection of the inserts highlighted areas of damage within the wear surface in both the CFR-PEEK and PEEK materials (Fig. 5). Further examination of these regions through scanning electron microscopy showed evidence of mechanical failure, with scratches and localised delamination apparent in both the PEEK and CFR-PEEK materials (Fig. 5). In the CFR-PEEK bearings there appeared to be deformation of the material, with a built-up region located on the posterior edge of the wear scar. In these regions, cracking and fracture of the material appeared visible, with suggestion of carbon-fibre pull-out as the fibre ends were clearly visible in some cases (Fig. 6). The PEEK inserts did not appear to show the material build-up in the same way, however localised delamination and large cracks were also apparent on the surface.

## 5 Discussion

Use of PEEK and CFR-PEEK as a substitute for UHMWPE in fixed bearing total knee replacements was investigated using custom-made flat inserts tested with a femoral bearing in current clinical use. Both materials exhibited very high wear rates under these test conditions, with large wear scars and evidence of mechanical material failure apparent. Slight differences in the shape of the wear scars on the PEEK and CFR-PEEK materials were noted across samples and between materials. It is likely that the lack of constraint, due to the flat insert, has resulted in some variability of the local kinematics at the femoral-insert interface between samples and stations. Conformity has been shown to have a significant influence on the wear performance of UHMWPE in total knee replacement, with lower conformity fixed bearings demonstrating low wear rates for both conventional and moderately cross-linked polyethylene. The wear rates within this study (252±159 mm^3^/Mc and 209±37 mm^3^/Mc for PEEK and CFR-PEEK respectively) were approximately 100 times higher than the wear of a flat geometry conventional polyethylene bearing articulating with the same femoral bearing design and under identical experimental simulator conditions (3.4±0.7 mm^3^/Mc) [9]. This wear behaviour may be considered unexpected in the context of experimental studies of CFR-PEEK in highly conforming total hip replacement and partial knee replacement [18], [20], [22], [29]. The exploration of these materials in a low conformity bearing, with higher contact pressure conditions, has not been previously undertaken.

Simple configuration pin on plate studies have demonstrated that UHMWPE exhibits reducing wear with increasing contact pressure [30], thus a low conformity fixed bearing knee replacement with lower contact area and higher contact pressure has been shown to have lower wear than a more conforming implant, when all other parameters were fixed [9]. Less research has been undertaken to explore the wear performance of PEEK and CFR-PEEK as bearings in total joint replacement, however, recent pin on plate studies have indicated increasing wear with increasing contact pressure, though this has not been demonstrated to be a significant trend [17], [31]. In addition to the implant geometry, material properties also influence the contact pressure within the knee replacement. The elastic modulus of PEEK and CFR-PEEK are higher than UHWMPE (4 GPa, 15 GPa and 1 GPa respectively). Application of simple Hertzian contact theory, for a static condition of 2.8 kN axial load applied to a total knee replacement in neutral position highlights the influence of material properties on the contact mechanics of the bearing [32]. Despite the loading conditions being identical, the predicted nominal contact pressure for a PEEK or CFR-PEEK flat insert was over an order of magnitude higher than an UHMWPE insert.

Analysis of the bearing surfaces following the wear study highlighted evidence of mechanical material failure, with delamination, deformation and cracking apparent in both the PEEK and CFR-PEEK materials. Additionally, there was evidence of carbon-fibre pull-out from the CFR-PEEK inserts, which may have contributed to the higher surface roughness observed on the metallic femoral bearings at the completion of the study. Such characteristics have not been previously observed in experimental studies of CFR-PEEK against a hard bearing in total joint replacement configurations, however wear through sub-surface cracking has been reported previously in sliding wear conditions with similar surface failure characteristics demonstrated [33]. Furthermore, a study investigating all-polymer bearings for cervical arthroplasty has also shown evidence of local CFR-PEEK delamination at the bearing surface, although not associated with the high wear observed in the present study [34]. Mechanical failure of PEEK has previously been reported, and therefore whilst the severity of damage observed and magnitude of wear may be high, it is not unexpected that PEEK is not a viable option for a total joint replacement whilst articulating with a metallic bearing [35]. Experimental studies of conventional and moderately cross-linked UHMWPE showed no such features, with only surface wear features such as polishing and burnishing previously reported [9], [28]. Indeed, the mechanical failure observed within the PEEK and CFR-PEEK bearings was more comparable with previously reported clinical failures in earlier knee replacement design [36].

There were some limitations within this study. Clearly the custom-made bearing materials were not representative of a currently used total knee replacement. The configuration was selected for two reasons, firstly the low conformity condition would create higher contact stress conditions and secondly, it was possible to compare with the lowest wear data generated within the research group [9]. As the study was conducted under displacement control, there is potential that the influence of the bearing design (such as the absence of natural constraint provided by a curved bearing) would produce differing results under a force-controlled study. Finally, to fully explore the performance of these novel materials in a total knee configuration, it would be advantageous to study the material under a range of kinematic conditions. However, the result achieved under a ‘standard’ test condition, when compared against existing data from polyethylene studies, provides a good insight into the material performance.

Experimental studies of PEEK have indicated no advantage when compared with UHMWPE in hard bearing configurations, and in many cases indicated PEEK to have poorer wear characteristics [13]. However, experimental studies of CFR-PEEK in hip and knee configurations to date have indicated CFR-PEEK to have comparable or better wear performance than UHMWPE [18], [37]. The present study appears to be the first to study these materials under adverse conditions and demonstrates the material may be an unsuitable bearing for a low conformity total knee replacement.

## 6 Conclusions

PEEK and CFR-PEEK materials were explored as an alternative for polyethylene in a low conformity knee replacement through an experimental wear study. Very high wear rates were observed for both materials with evidence of cracking and material failure observed within the wear region. It is likely the low conformity geometry resulted in high contact stress conditions caused localised mechanical failure of the material. This study demonstrates that neither material would be a suitable replacement for polyethylene in a low conformity total knee replacement articulating with a hard femoral bearing. The elevated wear observed with both materials, in contrast to previously reported total joint replacement studies, also highlights the need to study new materials in adverse condition arrangements, where contact stresses may become elevated and the tribological conditions sub-optimal.

## Open data

The data associated with this paper are openly available from the University of Leeds Data Repository [38].

## References

[1] Argenson J.-N. (2013). Survival analysis of total knee arthroplasty at a minimum 10 years' follow-up: a multicenter French nationwide study including 846 cases. Orthop. Traumatol.: Surg. Res..

[2] Steiger R.Nd. (2015). Lower prosthesis-specific 10-year revision rate with crosslinked than with non-crosslinked polyethylene in primary total knee arthroplasty: 386,104 procedures from the Australian orthopaedic association national joint replacement registry. Acta Orthop..

[3] Kurtz S. (2007). Projections of primary and revision hip and knee arthroplasty in the United States from 2005 to 2030. J. Bone Jt. Surg..

[4] Kurtz S. (2009). Future young patient demand for primary and revision joint replacement: national projections from 2010 to 2030. Clin. Orthop. Relat. Research®.

[5] Dalury D.F. (2013). Why are total knee arthroplasties being revised?. J. Arthroplast..

[6] Williams I.R., Mayor M.B., Collier J.P. (1998). The impact of sterilization method on wear in knee arthroplasty. Clin. Orthop. Relat. Res..

[7] Kurtz S.M. (1999). Advances in the processing, sterilization, and crosslinking of ultra-high molecular weight polyethylene for total joint arthroplasty. Biomaterials.

[8] Sathasivam S., Walker P. (1999). The conflicting requirements of laxity and conformity in total knee replacement. J. Biomech..

[9] Galvin A.L. (2009). Effect of conformity and contact stress on wear in fixed-bearing total knee prostheses. J. Biomech..

[10] A. Abdelgaied, et al., The effect of insert conformity and material on total knee replacement wear, Proc. Inst. Mech. Eng. Part H: J. Eng. Med., vol. 228(1), pp. 98–106, 2014. 10.1177/0954411913513251 PMC4361477 24297773

[11] Pruitt L.A. (2013). Clinical trade‐offs in cross‐linked ultrahigh‐molecular‐weight polyethylene used in total joint arthroplasty. J. Biomed. Mater. Res. Part B: Appl. Biomater..

[12] Atwood S.A. (2011). Tradeoffs amongst fatigue, wear, and oxidation resistance of cross-linked ultra-high molecular weight polyethylene. J. Mech. Behav. Biomed. Mater..

[13] S. Scholes and A.Unsworth, The wear properties of CFR-PEEK-OPTIMA articulating against ceramic assessed on a multidirectional pin-on-plate machine, Proc. Inst. Mech. Eng. Part H: J. Eng. Med., vol. 221(3), pp. 281–289, 2007. 10.1243/09544119JEIM224 17539583

[14] R.H. East, A.Briscoe, and A.Unsworth, Wear of PEEK-OPTIMA® and PEEK-OPTIMA®-wear performance articulating against highly cross-linked polyethylene, Proc. Inst. Mech. Eng. Part H: J. Eng. Med., vol. 229(3), pp. 187–193, 2015. 10.1177/0954411915576353 25833994

[15] Scholes S., Unsworth A. (2009). Wear studies on the likely performance of CFR-PEEK/CoCrMo for use as artificial joint bearing materials. J. Mater. Sci.: Mater. Med..

[16] Scholes S., Unsworth A. (2010). The wear performance of PEEK-OPTIMA based self-mating couples. Wear.

[17] A. Evans, et al., The influence of nominal stress on wear factors of carbon fibre–reinforced polyetheretherketone (PEEK-OPTIMA® Wear Performance) against zirconia toughened alumina (Biolox® delta ceramic), Proc. Inst. Mech. Eng. Part H: J. Eng. Med., p. 0954411914538783, 2014. 10.1177/0954411914538783 24898444

[18] Brockett C.L. (2012). Wear of ceramic‐on‐carbon fiber‐reinforced poly‐ether ether ketone hip replacements. J. Biomed. Mater. Res. Part B: Appl. Biomater..

[19] S. Flanagan, E.Jones, and C.Birkinshaw, In vitro friction and lubrication of large bearing hip prostheses, Proc. Inst. Mech. Eng. Part H: J. Eng. Med., vol. 224(7), pp. 853–864, 2010. 10.1243/09544119JEIM733 20839653

[20] S. Scholes, et al., Tribological assessment of a flexible carbon-fibre-reinforced poly (ether—ether—ketone) acetabular cup articulating against an alumina femoral head, Proc. Inst. Mech. Eng. Part H: J. Eng. Med., vol. 222(3), pp. 273–283, 2008. 10.1243/09544119JEIM334 18491697

[21] Grupp T.M. (2010). Biotribology of alternative bearing materials for unicompartmental knee arthroplasty. Acta Biomater..

[22] S. Scholes and A.Unsworth, Pitch-based carbon-fibre-reinforced poly (ether—ether—ketone) OPTIMA® assessed as a bearing material in a mobile bearing unicondylar knee joint, Proc. Inst. Mech. Eng. Part H: J. Eng. Med., vol. 223(1), pp. 13–25, 2009. 10.1243/09544119JEIM471 19239064

[23] ISO, Implants for Surgery – Wear of Total Knee-joint Prostheses Part 3: Loading and Displacement Parameters for Wear-testing Machines with Displacement Control and Corresponding Environmental Conditions for Test, 2004.

[24] McEwen H.M.J. (2005). The influence of design, materials and kinematics on the in vitro wear of total knee replacements. J. Biomech..

[25] Lafortune M.A. (1992). Three-dimensional kinematics of the human knee during walking. J. Biomech..

[26] P.I. Barnett, et al., Investigation of wear of knee prostheses in a new displacement/force-controlled simulator, Proc. Inst. Mech. Eng. Part H: J. Eng. Med., vol. 216(1), pp. 51–61, 2002. 10.1243/0954411021536289 11908483

[27] I.B. Solutions, Typical Material Properties datasheets for Optima and Motis, 2009.

[28] C. Brockett, et al., Influence material and geometry on the wear of fixed bearing total knee replacements, Bone Joint J. Orthop. Proc. Suppl., vol. 96(Suppl. 11), 2014, (p. 7-7).

[29] Grupp T. (2013). Biotribology of a new bearing material combination in a rotating hinge knee articulation. Acta Biomater..

[30] Barbour P.S.M., Barton D.C., Fisher J. (1997). The influence of stress conditions on the wear of UHMWPE for total joint replacements. J. Mater. Sci.-Mater. Med..

[31] Brockett C. (2016). PEEK AND CFR-PEEK AS ALTERNATIVE BEARING MATERIALS TO POLYETHYLENE. Bone Jt. J.

[32] Z.M. Jin, D.Dowson, J.Fisher, Contact pressure prediction in total knee joint replacements. Part 1: general elasticity solution for elliptical layered contacts, Proc. Inst. Mech. Eng. Part H - J. Eng. Med., vol. 209(1), pp. 1–8, 1995. 10.1243/PIME_PROC_1995_209_311_02 7669116

[33] Schroeder R. (2013). Failure mode in sliding wear of PEEK based composites. Wear.

[34] G.D.G. Langohr, H.A.Gawel, J.B.Medley, Wear performance of all-polymer PEEK articulations for a cervical total level arthroplasty system, Proc. Inst. Mech. Eng. Part H - J. Eng. Med., vol. 225(6), pp. 499–513, 2011.

[35] Grupp T.M. (2010). Alternative bearing materials for intervertebral disc arthroplasty. Biomaterials.

[36] Wright T.M. (1992). Wear of polyethylene in total joint replacements: observations from retrieved PCA knee implants. Clin. Orthop. Relat. Res..

[37] Wang Q.Q. (2012). Biotribological study of large diameter ceramic-on-CFR-PEEK hip joint including fluid uptake, wear and frictional heating. J. Mater. Sci.: Mater. Med..

[38] C. Brockett, et al., PEEK and CFR-PEEK Knee Wear Dataset, 2016. 10.5518/139.

